# Leukocytoclastic Vasculitis: Distinguishing Drug-Induced From Hepatitis C-Related Causes

**DOI:** 10.7759/cureus.80521

**Published:** 2025-03-13

**Authors:** Charles J DeBiase, Stephen K Stacey, Richard J LaBaere

**Affiliations:** 1 Family Medicine, A.T. Still University Kirksville College of Osteopathic Medicine, Kirksville, USA; 2 Family Medicine, Mayo Clinic Health System, La Crosse, USA

**Keywords:** autoimmune disease, cutaneous vasculitis, drug-induced cutaneous vasculitis, drug-induced reaction, hepatitis c (hcv) infection, hepatitis c management, leukocytoclastic vasculitis (lcv), palpable purpura, small vessel vasculitis

## Abstract

This report presents the case of a 34-year-old female with chronic cholecystitis and hepatic failure due to alcohol use and hepatitis C. She also has a history of opioid and alcohol use disorders. Frequent hospitalizations for fluid overload culminated in her presentation with lower extremity swelling and palpable purpura. Investigation revealed leukocytoclastic vasculitis, initially raising concern for mixed cryoglobulinemia from hepatitis C but ultimately more likely due to drug-induced vasculitis. This case highlights the diagnostic challenge of differentiating between hepatitis C-associated mixed cryoglobulinemia and drug-induced vasculitis, emphasizing the need for thorough evaluation to guide appropriate management.

## Introduction

Leukocytoclastic vasculitis (LCV) is an immune-mediated small vessel vasculitis characterized by neutrophilic infiltration and fibrinoid necrosis of postcapillary venules, often presenting as palpable purpura on the lower extremities. It can be triggered by infections, autoimmune conditions, or medications [1]. The global incidence of cutaneous LCV varies from 15 to 38 cases per million per year, depending on the definition used. The etiology remains unidentified in approximately half of the cases [2]. In our patient, the potential causes included her chronic hepatitis C infection or recent antibiotic use due to the recent addition of metronidazole and cefdinir, both of which are known triggers for vasculitis. Other common causes of drug-induced vasculitis to consider include NSAIDs, diuretics, and antithyroid medications [1]. Distinguishing between underlying etiologies is critical, as treatment varies depending on the cause. Here, we present a case of a 34-year-old female with chronic hepatic failure from alcohol use and hepatitis C who developed lower extremity palpable purpura, initially raising concern for cryoglobulinemia-associated vasculitis but ultimately determined to be likely drug-induced. This case underscores the need for careful evaluation in patients with vasculitic presentations to ensure accurate diagnosis and appropriate management.

## Case presentation

A 34-year-old female with hepatic failure secondary to alcohol use (currently in remission), untreated hepatitis C, and opioid use disorder presented to the emergency department with lower extremity swelling and palpable purpura that had begun earlier that day. She had been discharged the previous day after treatment for acute-on-chronic cholecystitis and was prescribed oral cefdinir and metronidazole upon discharge for continued antimicrobial coverage targeting biliary pathogens.

**Figure 1:**
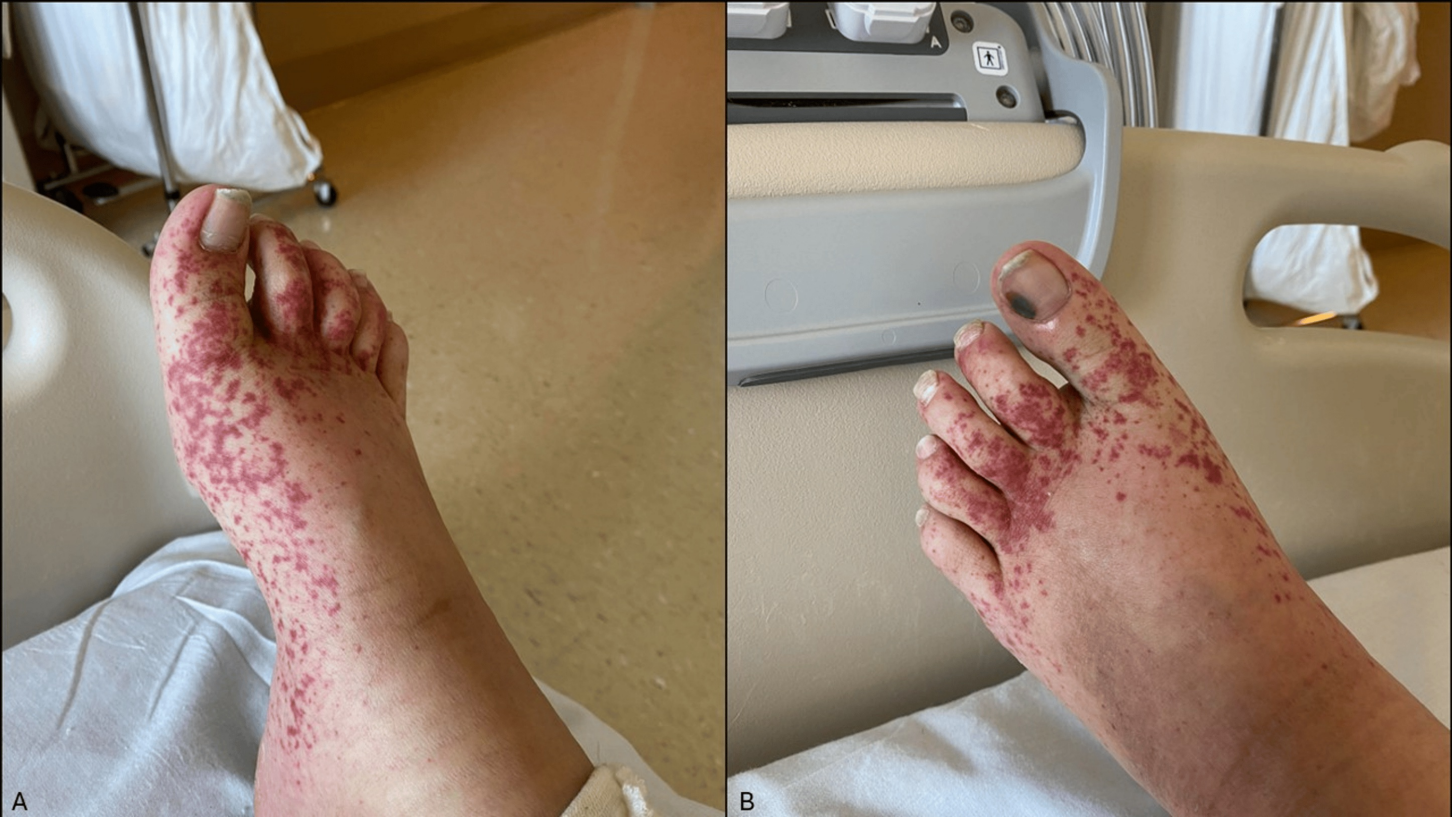
Images of the patient's right (A) and left (B) feet showing multiple palpable purpuric lesions, predominantly clustered on the dorsal surfaces.

On presentation, she reported ongoing right upper quadrant pain rated as a 7/10, consistent with her level of pain at discharge from the previous hospital stay. She denied shortness of breath, chest pain, fevers, chills, and nausea. Examination revealed jaundice and tender, palpable purpura clustered on her trunk and extremities, being the most severe on the dorsal feet (Figure 1). Right upper quadrant tenderness was also noted, consistent with her discharge examination. No other significant findings were observed.

In the emergency department, she had a temperature of 36.2°C, blood pressure of 137/75 mmHg, heart rate of 105 beats per minute, respirations of 18 breaths per minute, and oxygen saturation of 97%. Complete blood count, comprehensive metabolic panel, and labs were obtained to evaluate the likely etiology of her palpable purpuric lesions (Table 1). Findings were notable for anemia (hemoglobin 8.30 g/dL), thrombocytopenia (110,000 platelets per microliter), mildly elevated aspartate aminotransferase (62 U/L), and elevated direct bilirubin (8.9 mg/dL). Hepatitis C viral load was high (1,040,000 international units per milliliter). C-reactive protein, lactate, and complement levels were within normal limits. HIV, rheumatoid factor, and cryoglobulin serology testing were negative. Serum protein electrophoresis was negative. A biopsy of the lesions on her foot showed LCV.

**Table 1:** Key laboratory values during hospitalization AST, aspartate aminotransferase; ESR, erythrocyte sedimentation rate; CRP, C-reactive protein; C4, complement 4

Parameter	Normal range (units)	Patient results (on admission)
Hemoglobin	11.0-14.5 g/dL	8.3
Platelets	150-450 x 10⁹/L	110
AST	0-33 U/L	62
Direct bilirubin	0.1-0.3 mg/dL	8.9
ESR	0-20 mm/hr	12
CRP	0-10 mg/L	6.4
Lactate	0.5-2.2 mmol/L	2.1
C4	10-40 mg/dL	31
HIV	Negative	Negative
Hepatitis C viral load	Undetectable	1,040,000
Rheumatoid factor	<14 IU/mL	Negative
Cryoglobulin serology	Negative	Negative
Serum protein electrophoresis	Normal	Normal

Based on the patient’s palpable purpura and skin biopsy findings, the differential diagnosis included mixed cryoglobulinemia associated with her history of hepatitis C and drug-induced vasculitis. Despite high hepatitis C viral load, hepatitis C-induced vasculitis was less likely given the patient’s normal complement levels, negative cryoglobulin serology, and normal rheumatoid factor. Other small-vessel vasculitides, such as ANCA-associated vasculitis or IgA vasculitis, were considered; however, the absence of systemic symptoms (e.g., renal involvement, pulmonary findings, or gastrointestinal symptoms) and negative serologies made these less likely. The temporal association between symptom onset and the initiation of new antibiotics strongly suggested drug-induced vasculitis as the most probable cause.

She was observed for another night. Due to the absence of systemic involvement, she was deemed safe for discharge in stable condition with plans for outpatient observation of her LCV and continued treatment with metronidazole and cefdinir. Due to the mild severity of her symptoms, steroid treatment was not initiated. One week later, she began outpatient treatment with sofosbuvir/velpatasvir for her hepatitis C infection, and her purpuric lesions had started to resolve. At three months post-treatment, her hepatitis C viral load was undetectable. At this time, her purpuric lesions had resolved. After an additional 36 months of observation, the patient has not experienced recurrence of her LCV.

## Discussion

LCV refers to a form of small vessel vasculitis primarily affecting postcapillary venules that can result from a variety of triggers, including infections, autoimmune diseases, and medications. In this case, the patient's chronic hepatitis C infection, combined with recent antibiotic use (metronidazole and cefdinir), presented a differential diagnosis between cryoglobulinemic vasculitis and drug-induced vasculitis. Given the overlap in clinical presentations, careful evaluation of her medical history and laboratory findings was essential for identifying the most likely etiology.

When hepatitis C is the underlying cause, the direct trigger is typically cryoglobulinemic vasculitis. Cryoglobulinemic vasculitis is a type of small vessel vasculitis characterized by the deposition of cryoglobulins in the blood vessels. Mixed cryoglobulinemia is the most prevalent form, accounting for approximately 90% of cases [3]. Among these, 70-90% of patients have concurrent hepatitis C infection. The cryoglobulins involved are immune complexes consisting of polyclonal IgG, antigens, and monoclonal or polyclonal IgM, which precipitate in the cold and dissolve upon warming [3,4]. Symptoms of mixed cryoglobulinemia can range from insidious to life-threatening, with varying prevalence of symptomatic cases across populations (2-50%) [5].

Drug-induced vasculitis is another common cause of LCV. A retrospective study involving 239 patients from Northern Spain identified nonsteroidal anti-inflammatory drugs and antibiotics as the most common triggers of drug-induced vasculitis [6]. Other medications implicated include methotrexate, cilastatin, and zidovudine, and others [7].

The primary clinical manifestation of LCV is palpable purpura [2]. Mixed cryoglobulinemia typically presents with a classic triad of arthralgia, palpable purpura, and fatigue in 30-80% of cases [8,9]. Multiple organ involvement can occur, including glomerulonephritis, peripheral neuropathy, hematological abnormalities, endocrine alterations, and hepatic issues [8].

Diagnosis is based on clinical features and laboratory findings. No single test can distinguish drug-induced vasculitis from other vasculitides [10]. In cases of suspected mixed cryoglobulinemic vasculitis, relevant laboratory findings include serology for cryoglobulinemia, elevated rheumatoid factor (nearly 100% of cases), decreased C4 levels (seen in approximately 90% of cases), and anti-hepatitis C virus antibodies, as hepatitis C is a major underlying trigger. These findings support an HCV-related etiology rather than drug-induced vasculitis, which typically lacks cryoglobulins and complement abnormalities [5,8]. Renal biopsy may be considered for suspected renal involvement, while skin biopsy, though not mandatory, can aid in atypical presentations. Skin biopsy for cryoglobulinemia is characteristic of LCV [11]. Histological features include neutrophilic infiltration, leukocytoclasia, fibrinoid necrosis, and vessel wall damage [2].

The severity of the disease guides treatment. Mild cases, with non-ulcerative cutaneous lesions and no life-threatening organ involvement, can be managed with analgesics and possibly low-dose glucocorticoids. Severe cases may require high-dose glucocorticoids plus rituximab [5,8,9]. In patients with hepatitis C, antiviral therapy is recommended and generally associated with improved outcomes [8]. For drug-induced vasculitis, while there is no standard treatment, the first step is often discontinuing the offending agent [10].

## Conclusions

Understanding the clinical presentation and appropriate diagnostic workup for cryoglobulinemic and drug-induced vasculitis are crucial for effective management. Our patient’s presentation of palpable purpura, confirmed by skin biopsy as LCV, highlighted the importance of assessing disease severity to guide treatment decisions. The absence of cryoglobulinemia and the normal rheumatoid factor and C4 levels made mixed cryoglobulinemia less likely and supported drug-induced vasculitis as the more probable diagnosis. Given her mild disease, characterized by non-ulcerative cutaneous lesions and no life-threatening organ involvement, it was deemed safe to continue her antibiotics with close outpatient monitoring for recurrence or persistence of vasculitis. Her subsequent successful antiviral treatment and lack of further episodes underscore the favorable prognosis in well-managed cases of mild vasculitis.
